# Disability-related inequalities in the prevalence of loneliness across the lifespan: trends from Australia, 2003 to 2020

**DOI:** 10.1186/s12889-024-17936-w

**Published:** 2024-02-27

**Authors:** Glenda M. Bishop, Gwynnyth Llewellyn, Anne M. Kavanagh, Hannah Badland, Jodie Bailie, Roger Stancliffe, Eric Emerson, Nicola Fortune, Zoe Aitken

**Affiliations:** 1 Melbourne School of Population and Global Health, The University of Melbourne, Melbourne, VIC 3010 Australia; 2 Centre for Disability Research and Policy, The University of Sydney, Camperdown, NSW 2006 Australia; 3 Social and Global Studies Centre, RMIT University, Melbourne, VIC 3000 Australia; 4 University Centre for Rural Health, The University of Sydney, Lismore, NSW 2480 Australia; 5 Centre for Disability Research, Faculty of Health & Medicine, Lancaster University, Lancaster, LA1 4YW UK; 6 College of Nursing and Health Sciences, Flinders University, Bedford Park, SA 5042 Australia

**Keywords:** Disability, Loneliness, Inequality, Time series

## Abstract

**Background:**

Experiencing loneliness can be distressing and increasing evidence indicates that being lonely is associated with poor physical and mental health outcomes. Cross-sectional studies have demonstrated that people with disability have increased risk of experiencing loneliness compared to people without disability. However, we do not know if these inequalities have changed over time. This study investigated the prevalence of loneliness for people with disability in Australia annually from 2003 to 2020 to examine whether disability-related inequalities in loneliness have changed over time, and disaggregated results for subgroups of people with disability by age group, sex, and disability group.

**Methods:**

We used annual data (2003–2020) from the Household, Income and Labour Dynamics in Australia Survey. Loneliness was measured by a single question assessing the subjective experience of loneliness. For each wave, we calculated population-weighted age-standardised estimates of the proportion of people experiencing loneliness for people with and without disability. We then calculated the absolute and relative inequalities in loneliness between people with and without disability for each wave. Analyses were stratified by 10-year age groups, sex, and disability group (sensory or speech, physical, intellectual or learning, psychological, brain injury or stroke, other).

**Results:**

From 2003 to 2020, the prevalence of loneliness was greater for people with disability, such that people with disability were 1.5 to 1.9 times more likely to experience loneliness than people without disability. While the prevalence of loneliness decreased for people without disability between 2003 and 2020, the prevalence of loneliness did not decrease for people with disability during this period. Inequalities in loneliness were more substantial for people with intellectual or learning disabilities, psychological disability, and brain injury or stroke.

**Conclusion:**

This study confirms that people with disability have increased risk of loneliness compared to people without disability. We add to the existing evidence by demonstrating that disability-related inequalities in loneliness have persisted for two decades in Australia without improvement. Our findings indicate that addressing inequalities in loneliness for people with disability is a critical public health concern given that loneliness is associated with a wide range of poor health outcomes.

**Supplementary Information:**

The online version contains supplementary material available at 10.1186/s12889-024-17936-w.

## Introduction

Loneliness is an unpleasant subjective experience that results from a perceived discrepancy between one’s desired and actual levels of satisfying social relationships [1] and serves as a signal of unmet human need [2], evoking craving responses for social interactions similar to those for hunger [3]. While loneliness is a common experience, prolonged and problematic levels of loneliness are not equally distributed in the population [4]. A recent conceptual model of loneliness by Lim and colleagues [5] proposes that loneliness occurs when a trigger, such as a significant life event or life stage transition, interacts with various underlying risk factors including socio-demographic factors (e.g., age, gender, marital status, migration status, living status, socio-economic status), personal health (e.g., physical, mental, cognitive), and socio-environmental factors (e.g., workplace, digital communication). Experiencing loneliness can be distressing and cause considerable suffering [6], however increasing evidence indicates that being lonely is also associated with poor health outcomes including increased mortality, increased risk of cardiovascular disease and metabolic syndrome, and poorer mental and emotional health [7–10]. The significant health issues associated with loneliness, together with the social factors that underpin it, mean that addressing loneliness requires a public health approach to understand the distribution of loneliness in the population and its impact on population health [4, 5, 8, 9, 11, 12].

One population group that experiences many of the risk factors for loneliness is people with disability. Many of these risk factors are structural in nature. For example, people with disability are more likely than people without disability to have lower education, lower income, be unemployed or underemployed, and live in vulnerable housing or within care homes or institutions, which lead to barriers to participation in various life domains [13–15]. People with disability also experience poorer health outcomes than people without disability [13, 15, 16], increasing their risk of loneliness. Furthermore, people with disability have less social support and are more socially isolated than people without disability [17, 18], which reduces their access to potential solutions that may attenuate loneliness, including relationships and community support [5]. It is therefore not surprising that people with disability are more likely to experience loneliness than people without disability [17, 19–23].

Although we know that people with disability experience inequalities in loneliness relative to people without disability, we do not know if the magnitude of inequality has changed over time. This evidence gap can be closed by tracking disability-related inequalities in loneliness over the last two decades using longitudinal population survey data. This knowledge is important given that loneliness is associated with poorer health outcomes [7–10]. Since people with disability have considerable health inequalities relative to people without disability [15, 16], and both loneliness and health inequalities are at least in part driven by the social determinants of health (e.g., employment, education) [5, 15], a clear understanding of the extent of inequalities in loneliness experienced by people with disability relative to people without disability is needed, as well as how the inequalities have changed in recent years. This evidence may lead to the development of policy solutions to address this issue and reduce the impact that inequalities in loneliness may have on the health inequalities experienced by people with disability relative to people without disability.

Disability is a complex biopsychosocial phenomenon resulting from dynamic interaction between biological, personal, and environmental factors, which together affect accessibility and participation within society [24]. The diverse nature of disability means that while it is important to examine changes in loneliness prevalence for people with disability compared to people without disability, it is also necessary to consider changes in loneliness prevalence for subgroups of people with disability. For instance, stratifying analyses by factors such as age, sex, and disability group (based on type of impairment, i.e., sensory or speech, physical, intellectual or learning, psychological, brain injury or stroke, other), allows examination of inequalities in loneliness in subgroups of the population of people with disability.

Therefore, this study investigated the prevalence of loneliness for people with disability in Australia and examined whether inequalities in loneliness for people with disability have changed over time. We used data from the longitudinal Household, Income and Labour Dynamics in Australia (HILDA) Survey to examine population-level changes in annual prevalence of loneliness for people with and without disability between 2003 and 2020 and further stratified the analysis by age, sex, and disability group.

## Methods

### Data source

The HILDA Survey is a longitudinal survey of Australian households, conducted annually since 2001 using in person interviews and self-completed questionnaires. Interviews are conducted with all household members aged 15+ years. In 2001, the original sample was a random national probability sample of private dwellings and included 7682 responding households with 13,969 respondents, with a household response rate of 66% [25]. Later waves of the survey included all participants from the original sample, with the addition of any children born into or adopted into the household, and new household members. In 2011, a top-up sample of 2153 households and 4009 respondents was added to maintain representativeness, with a top-up household response rate of 69% [25]. Over the first 20 waves, 33,347 participants were interviewed. Individual response rates for continuing participants were approximately 95% across the waves [26]. Additional details about the HILDA Survey have been published [26, 27]. This study used General Release 20 of the HILDA Survey, which included 20 annual waves from 2001 to 2020 [28].

### Disability measures

During every wave, participants were asked “do you have any long-term health condition, impairment or disability (such as these) that restricts you in your everyday activities, and has lasted or is likely to last, for 6 months or more?” Flashcards were shown to the respondents during their interview to provide examples of impairments, disabilities or conditions that broadly align with the International Classification of Functioning, Disability and Health (ICF) conceptualisation of disability [24]. The flashcards were updated in 2003 to provide a broader range of conditions, thus we analysed data from 2003 onwards to ensure consistency in the definition of disability that was used for each wave.

We classified participants with disability into six disability groups: sensory or speech (sight problems not corrected by glasses; hearing problems; speech problems), physical (including blackouts; difficulty gripping things; limited use of legs or feet and restricted activities due to chronic pain), intellectual or learning (difficulty learning or understanding things), psychological (mental illness; nervous or emotional condition), brain injury or stroke (long term effects resulting from head injury, stroke or other brain damage), other/type not specified (other long-term conditions that restricted everyday activities or impairment types which were not otherwise specified). The choice of these six categories is based on those used to report data from the Survey of Disability, Ageing and Carers (SDAC) [29], the most comprehensive source of data on disability prevalence in Australia. The six SDAC disability groups are constructed based on underlying health conditions, impairments, activity limitations and participation restrictions. However, due to the phrasing of disability questions within the HILDA Survey, we were unable to match the SDAC group for ‘psychosocial’; we therefore used the disability group definition for ‘psychological’, which is consistent with the SDAC grouping prior to 2015 but slightly narrower than the disability group termed ‘psychosocial’ used in SDAC from 2015 onwards [30]. The alignment between the HILDA Survey variables and the six disability groups used by SDAC for reporting purposes has been published elsewhere [31 (Table 1 of the Supplementary Materials)]. Note that people with disability can belong to more than one disability group, thus the sum of prevalence across disability groups can exceed 100%.

### Loneliness measure

Loneliness was measured in every wave by asking participants how much they agreed or disagreed with the statement “I often feel very lonely”, using a Likert scale ranging from 1 (strongly disagree) to 7 (strongly agree). This directly assesses the subjective experience of the participant in relation to feelings of loneliness, separate to concepts associated with social connectedness. Loneliness scores followed a strongly right-skewed non-normal distribution; thus, we chose not to analyse loneliness scores as a continuous measure. Instead, a dichotomised measure was generated for any type of agreement to the question, such that a score of 5–7 was designated as ‘experiencing loneliness’, consistent with other studies [32–34].

### Age standardisation

Given that age is related to both disability [35] and loneliness [e.g., 36], it is likely to be an important confounder of estimates of differences in loneliness between people with and without disability. To adjust for this, we used direct age standardisation. We chose to standardise to the age distribution of people with disability in the most recent wave (2020) of the HILDA survey, applying 5-year age groups that were top-coded at 85+ years. This approach was chosen because the age distribution of people with disability is substantially different to the national population, given that the prevalence of disability increases substantially after 65 years [35], resulting in older people being over-represented in the disability subpopulation. This approach to standardisation prevents obscuring inequalities because the disability group loneliness estimates used are the actual age-specific prevalence estimates for people with disability, reflecting the reality for the population of interest [37, 38].

### Statistical analysis

This study used eighteen waves of data from 2003 to 2020. The analytic sample consisted of 278,057 observations, obtained from 31,040 participants aged 15+ years who responded to the disability question (99.9% of respondents). To describe the Australian population aged 15+ years with and without disability, we conducted population-weighted descriptive analyses for the first and last wave contributing to the analysis (2003 and 2020) to assess the distribution of age (based on the participant age on 30 June for each wave), sex and disability group. We used survey weights provided by HILDA and the Taylor Series linearisation method for standard error calculation to adjust for clustering and stratification in the survey design and non-response [39].

To compare the prevalence of loneliness in people with and without disability for each wave of the HILDA Survey, we calculated population-weighted age-standardised estimates of the proportion of people who reported experiencing loneliness. It is important to note that age standardisation results in estimates of prevalence of loneliness for people with disability that are largely unadjusted, while estimates for people without disability provide hypothetical prevalence estimates that would have been observed had people without disability had the same age profile as the 2020 disabled population. In this way, the percentages presented can be meaningfully compared for people with and without disability, knowing that any differences are not due to different population age structures. We then calculated the absolute and relative inequalities in loneliness between people with and without disability for each wave. The absolute inequality in loneliness was calculated by subtracting the prevalence of loneliness in people without disability from the prevalence of loneliness in people with disability. The relative inequality in loneliness was calculated by dividing the prevalence of loneliness in people with disability by the prevalence of loneliness in people without disability. Analyses were repeated on the data following disaggregation by 10-year age groups and/or sex, and by disability group. To detect trends between 2003 and 2020 in the prevalence, absolute inequalities or relative inequalities in loneliness between people with and without disability, we assessed each measure for the presence of non-overlapping 95% confidence intervals between the start and end years.

Stata SE version 17.0 was used to perform all statistical analyses. Graphs were generated using R version 4.2.1, RStudio version 2022.07.1 and ggplot2 version 3.3.6.

### Missing data

Overall, 11.2% of observations had missing data for the loneliness question, but no values were missing for age, sex or disability. Analysis of the missing loneliness data, when stratified by disability, demographic and socio-economic characteristics suggested that the data were not missing completely at random (Table S1, Supplementary Material), but could be missing at random (i.e., likelihood of missingness was conditional on the observed data but not the missing data). Based on Lee and colleagues framework for the handling of missing data in observational studies [40], complete case analysis was likely to be biased given that the probability of missingness of the outcome measure was expected to depend on the outcome or exposure. Thus, to reduce potential bias we performed multiple imputation using predicted mean matching with 50 imputations (see Supplementary Material for further details).

## Results

### Population characteristics

The population-weighted demographic characteristics for the first and final waves, stratified by disability, are shown in Table 1. The population-weighted prevalence of disability changed little between 2003 and 2020, with a prevalence of 27.8% (95% CI: 26.6%, 29.1%) in 2003 and 29.3% (95% CI: 28.0%, 30.6%) in 2020. People with disability were more likely to be in older age groups, with a mean age in 2003 of 53.6 years (95% CI: 52.6,54.6) and in 2020 of 55.1 years (95% CI: 54.2, 56.0). In contrast, for people without disability, the mean age in 2003 was 39.6 years (95% CI: 39.1, 40.2) and in 2020 was 41.6 years (95% CI: 41.1, 42.1). There was little difference in the proportions of males and females between people with and without disability.

**Table 1:** Population-weighted estimates of demographic characteristics, and disability groups, for people with and without disability in 2003 and 2020

	2003		2003		2020		2020	
	Any disability		No disability		Any disability		No disability	
Characteristic	(*n* = 3553)		(*n* = 9175)		(*n* = 5112)		(*n* = 11,953)	
	%	95% CI	%	95% CI	%	95% CI	%	95% CI
Age (years)								
15–24	7.8	(6.8, 9.0)	21.5	(20.2, 22.9)	9.6	(8.3, 11.0)	18.0	(16.9, 19.1)
25–34	10.3	(9.1, 11.7)	21.1	(19.8, 22.5)	10.4	(9.4, 11.6)	21.6	(19.5, 23.8)
35–44	14.0	(12.8, 15.4)	21.0	(19.9, 22.1)	9.1	(8.1, 10.2)	19.9	(18.3, 21.5)
45–54	17.2	(15.6, 18.9)	17.5	(16.5, 18.6)	15.1	(13.4, 17.0)	16.0	(14.9, 17.2)
55–64	19.5	(17.8, 21.3)	10.0	(9.2, 10.9)	18.7	(17.3, 20.1)	12.7	(11.8, 13.3)
65–74	15.3	(13.8, 16.9)	6.0	(5.3, 6.8)	19.5	(17.9, 21.1)	8.1	(7.3, 9.0)
75+	15.9	(13.9, 18.0)	2.8	(2.4, 3.3)	17.6	(16.1, 19.3)	3.8	(3.3, 4.3)
Sex								
Male	50.1	(48.4, 51.9)	48.9	(47.8, 50.0)	47.2	(45.5, 48.8)	49.8	(48.6, 51.1)
Female	49.9	(48.1, 51.6)	51.1	(50.0, 52.2)	52.8	(51.2, 54.5)	50.2	(48.9, 51.4)
Disability group ^a^								
Sensory or speech	24.1	(22.5, 25.7)			21.8	(20.2, 23.5)		
Physical	56.8	(54.7, 58.9)			60.7	(58.6, 62.9)		
Intellectual or learning	3.1	(2.4, 4.1)			6.8	(5.8, 8.0)		
Psychological	10.8	(9.5, 12.2)			21.4	(19.9, 23.1)		
Brain injury or stroke	3.6	(2.9, 4.6)			4.7	(3.9, 5.6)		
Other/type not specified	55.9	(53.8, 58.0)			59.1	(57.1, 61.0)		

^a^ Only available for people with disability. Note that people may have multiple disabilities and belong to more than one disability group, thus the percentages do not tally to 100

Among people with disability, the most common disability group was physical, followed by sensory or speech, and psychological. While the proportion of people with disability who had physical disability or sensory or speech disability remained stable between 2003 and 2020, the proportion of people with psychological disability almost doubled from 10.8 to 21.4%. A similar increase was seen in the proportion of people with intellectual or learning disability, which increased from 3.1% in 2003 to 6.8% in 2020.

### Loneliness prevalence and inequalities by disability

**Fig. 1:**
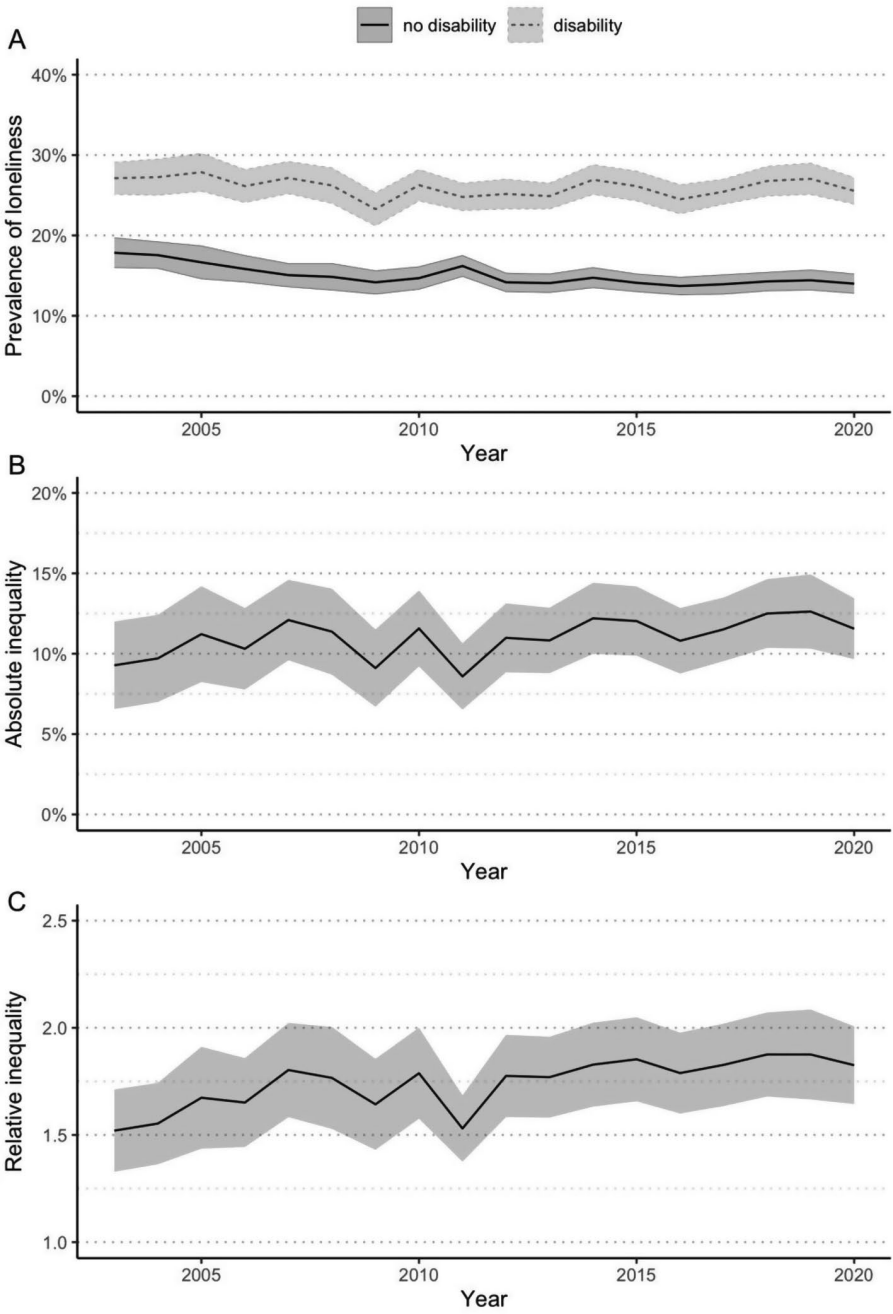
Loneliness over time for Australians aged 15+ years with and without disability, 2003–2020. (**A**) Age-standardised population-weighted prevalence of loneliness over time, with 95% confidence intervals. Absolute (**B**) and relative (**C**) inequalities in the prevalence of loneliness over time, with 95% confidence intervals

The population-weighted age-standardised estimates of the prevalence of loneliness for the first and final waves (2003 and 2020), and the absolute and relative inequalities between people with and without disabilities, are shown in Table 2. Figure 1A shows that across all 18 waves, the prevalence of loneliness was higher for people with disability than for people without disability. Furthermore, the prevalence of loneliness for people with disability remained stable over time with a prevalence of 27.1% in 2003 and 25.6% in 2020, where the 95% confidence intervals for each year overlapped. In contrast, the prevalence of loneliness for people without disability decreased from 17.8% in 2003 to 14.0% in 2020, with the decrease occurring between 2003 and 2009. Figure 1B demonstrates that the absolute inequalities in loneliness between people with and without disability was consistent across all waves with no obvious time trends. In contrast, Fig. 1C demonstrates that when relative inequalities were considered, people with disability in 2003 were 1.52 times more likely to experience loneliness than people without disability, and in 2020 were 1.83 times more likely to experience loneliness. While this change suggests an increasing trend, driven by the decrease in prevalence of loneliness in people without disability but not for people without disability, the 95% confidence intervals of the relative inequalities overlapped. The largest relative inequality was observed in 2015 and 2018, where people with disability were 1.88 times more likely to experience loneliness.

**Table 2:** Population-weighted age-standardised estimates of the prevalence of loneliness, absolute inequalities and relative inequalities for people with disability when compared to people without disability, in 2003 and 2020

	Prevalence		Prevalence		Absolute inequality ^a^		Absolute inequality ^a^		Relative inequality ^a^		Relative inequality ^a^	
	2003		2020		2003		2020		2003		2020	
	%	95% CI	%	95% CI	%	95% CI	%	95% CI	Ratio	95% CI	Ratio	95% CI
Whole population												
Disability	27.1	(25.1, 29.1) ^b^	25.6	(23.9, 27.2) ^b^	9.3	(6.6, 12.0)	11.6	(9.7, 13.5)	1.52	(1.33, 1.71)	1.83	(1.64, 2.01)
No disability	17.8	(16.0, 19.7)	14.0	(12.8, 15.2) ^c^								
Age 15–24 years												
Disability	28.7	(22.1, 35.4) ^b^	39.7	(33.6, 45.7) ^b^	10.1	(3.1, 17.0)	16.5	(10.0, 23.0)	1.54	(1.14, 1.94)	1.71	(1.39, 2.03)
No disability	18.6	(16.1, 21.2)	23.2	(20.4, 26.0)								
Age 25–34 years												
Disability	27.7	(21.3, 34.1) ^b^	33.0	(28.1, 37.9) ^b^	9.7	(2.7, 16.7)	16.1	(10.4, 21.7)	1.54	(1.11, 1.97)	1.95	(1.52, 2.37)
No disability	17.9	(15.1, 20.7)	16.9	(14.3, 19.6)								
Age 35–44 years												
Disability	28.8	(24.0, 33.5) ^b^	31.7	(25.6, 37.7) ^b^	11.1	(6.0, 16.1)	16.8	(10.5, 23.0)	1.63	(1.30, 1.95)	2.12	(1.62, 2.62)
No disability	17.7	(15.3, 20.1)	14.9	(12.5, 17.3)								
Age 45–54 years												
Disability	26.2	(21.9, 30.5) ^b^	25.2	(20.9, 29.6) ^b^	10.6	(5.7, 15.5)	11.4	(6.8, 16.0)	1.68	(1.31, 2.04)	1.83	(1.44, 2.21)
No disability	15.6	(13.4, 17.8)	13.8	(11.8, 15.8)								
Age 55–64 years												
Disability	27.5	(23.0, 32.0) ^b^	24.7	(21.3, 28.2) ^b^	13.0	(7.5, 18.5)	13.6	(9.7, 17.4)	1.90	(1.39, 2.41)	2.21	(1.74, 2.68)
No disability	14.5	(11.4, 17.6)	11.2	(9.3, 13.0)								
Age 65–74 years												
Disability	22.8	(18.5, 27.1)	17.8	(14.7, 20.8) ^b^	3.8	(-2.6, 10.2)	7.6	(3.4, 11.8)	1.20	(0.82, 1.58)	1.75	(1.14, 2.35)
No disability	19.0	(14.1, 24.0)	10.2	(6.8, 13.5) ^c^								
Age 75+ years												
Disability	29.8	(24.4, 35.2)	19.8	(16.2, 23.4) ^c^	8.6	(-0.6, 17.9)	5.8	(0.6, 11.0)	1.41	(0.85, 1.96)	1.41	(0.96, 1.87)
No disability	21.2	(13.9, 28.4)	14.0	(10.2, 17.8)								
Male												
Disability	25.3	(22.6, 27.9) ^b^	23.8	(21.4, 26.2) ^b^	9.5	(5.7, 13.4)	11.2	(8.5, 14.0)	1.61	(1.28, 1.94)	1.90	(1.61, 2.18)
No disability	15.7	(12.9, 18.5)	12.5	(11.0, 14.1)								
Female												
Disability	28.9	(26.2, 31.7) ^b^	27.3	(25.1, 29.5) ^b^	9.5	(5.9, 13.2)	11.9	(9.3, 14.5)	1.49	(1.26, 1.72)	1.78	(1.55, 2.00)
No disability	19.4	(17.2, 21.6)	15.3	(13.7, 17.0) ^c^								
Disability group												
Sensory or speech	26.6	(22.4, 30.8) ^b^	26.8	(22.9, 30.7) ^b^	8.8	(4.2, 13.4)	12.8	(8.6, 16.9)	1.49	(1.21, 1.77)	1.91	(1.58, 2.24)
Physical	29.9	(27.1, 32.7) ^b^	26.2	(24.2, 28.2) ^b^	12.1	(8.7, 15.5)	12.2	(9.9, 14.4)	1.68	(1.44, 1.91)	1.87	(1.66, 2.07)
Intellectual or learning	28.5	(16.8, 40.2)	39.1	(31.1, 47.2) ^b^	10.6	(-1.2, 22.4)	25.2	(17.1, 33.2)	1.60	(0.93, 2.27)	2.80	(2.19, 3.41)
Psychological disability	45.5	(38.1, 52.9) ^b^	36.5	(32.5, 40.5) ^b^	27.7	(20.0, 35.3)	22.5	(18.3, 26.6)	2.55	(2.06, 3.05)	2.61	(2.25, 2.99)
Brain injury or stroke	41.5	(31.0, 52.0) ^b^	39.7	(31.8, 47.6) ^b^	23.7	(13.1, 34.3)	25.7	(17.7, 33.7)	2.33	(1.70, 2.95)	2.83	(2.22, 3.45)
Other/type not specified	28.0	(25.5, 30.6) ^b^	25.7	(23.5, 27.9) ^b^	10.2	(7.2, 13.2)	11.7	(9.4, 14.1)	1.57	(1.36, 1.78)	1.84	(1.63, 2.05)
No disability	17.8	(16.0, 19.7)	14.0	(12.8, 15.2) ^c^								

^a^ The values reported represent the absolute or relative inequalities derived from comparing to the ‘no disability’ group within that subpopulation

^b^ Non-overlapping 95% confidence intervals when compared to ‘no disability’ group within that subpopulation

^c^ Non-overlapping 95% confidence intervals when compared to the same subpopulation in 2003

### Loneliness prevalence and inequalities by disability, age and sex

**Fig. 2:**
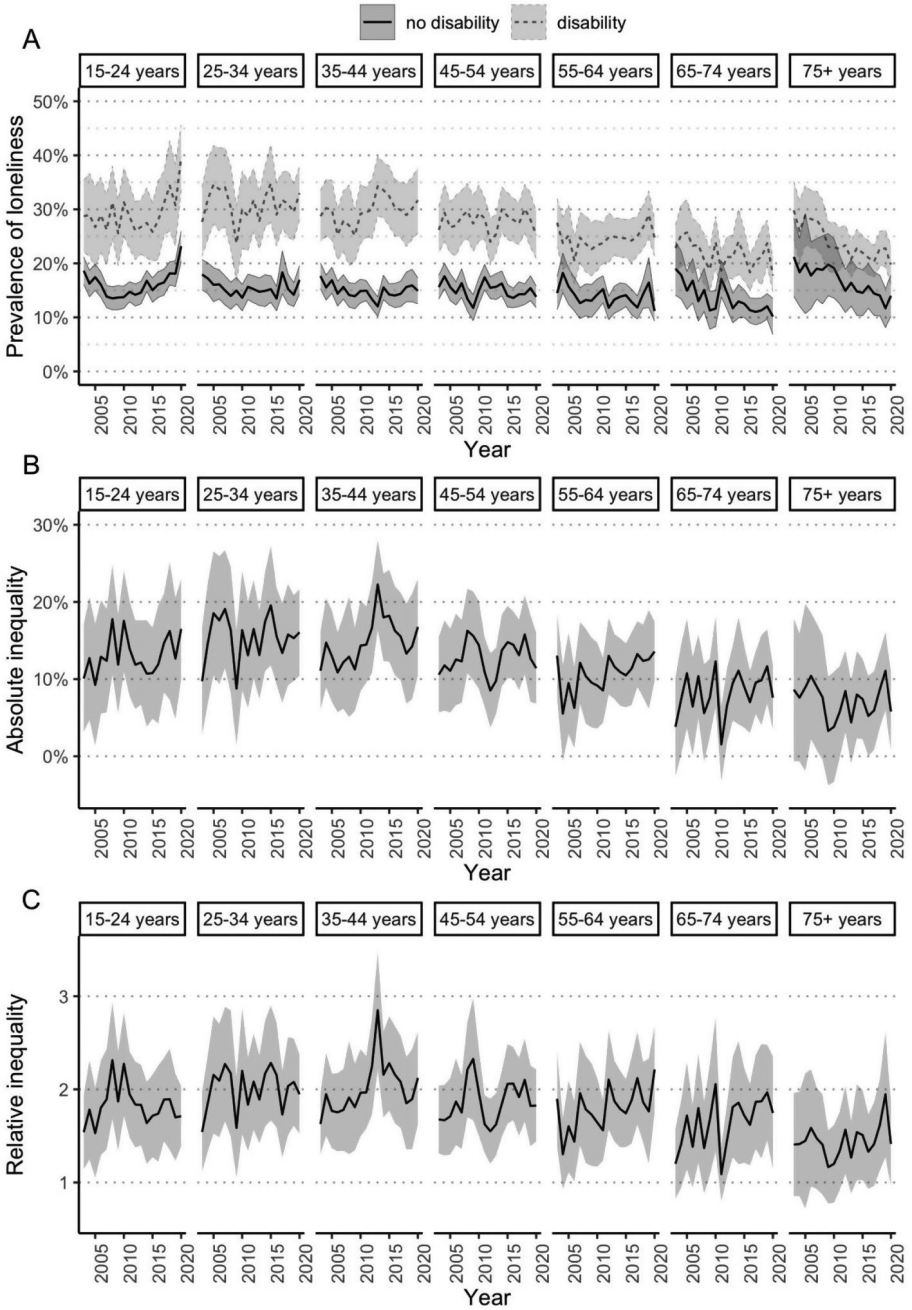
Loneliness over time for Australians aged 15+ years with and without disability, 2003–2020, stratified by age group. (**A**) Age-standardised population-weighted prevalence of loneliness over time, stratified by age group, with 95% confidence intervals. Absolute (**B**) and relative (**C**) inequalities in the prevalence of loneliness over time, stratified by age group, with 95% confidence intervals

Figure 2A demonstrates that when stratified by age group, the prevalence of loneliness was higher for people with disability than for people without disability in every 10-year age group between 15 and 64 years. When comparing trends over time, the prevalence of loneliness remained stable over the 18-year period for people aged between 25 and 64 years, for both people with and without disability. There was a trend toward increasing prevalence of loneliness in people with disability aged 15–24 years between 2003 and 2020, mostly occurring between 2015 and 2020, however the 95% confidence intervals overlapped. In contrast, for people with disability aged 75+ years, the prevalence of loneliness decreased from 29.8% in 2003 to 19.8% in 2020. For people without disability, there was little difference in the prevalence of loneliness by age group or between waves. Both the absolute and relative inequalities in loneliness between people with and without disability (Fig. 2B and C), stratified by age group, remained stable between 2003 and 2020. While the inequalities tended to be smaller for people aged 65+ years, the 95% confidence intervals overlapped when compared to the inequalities for people aged under 65 years.

**Fig. 3:**
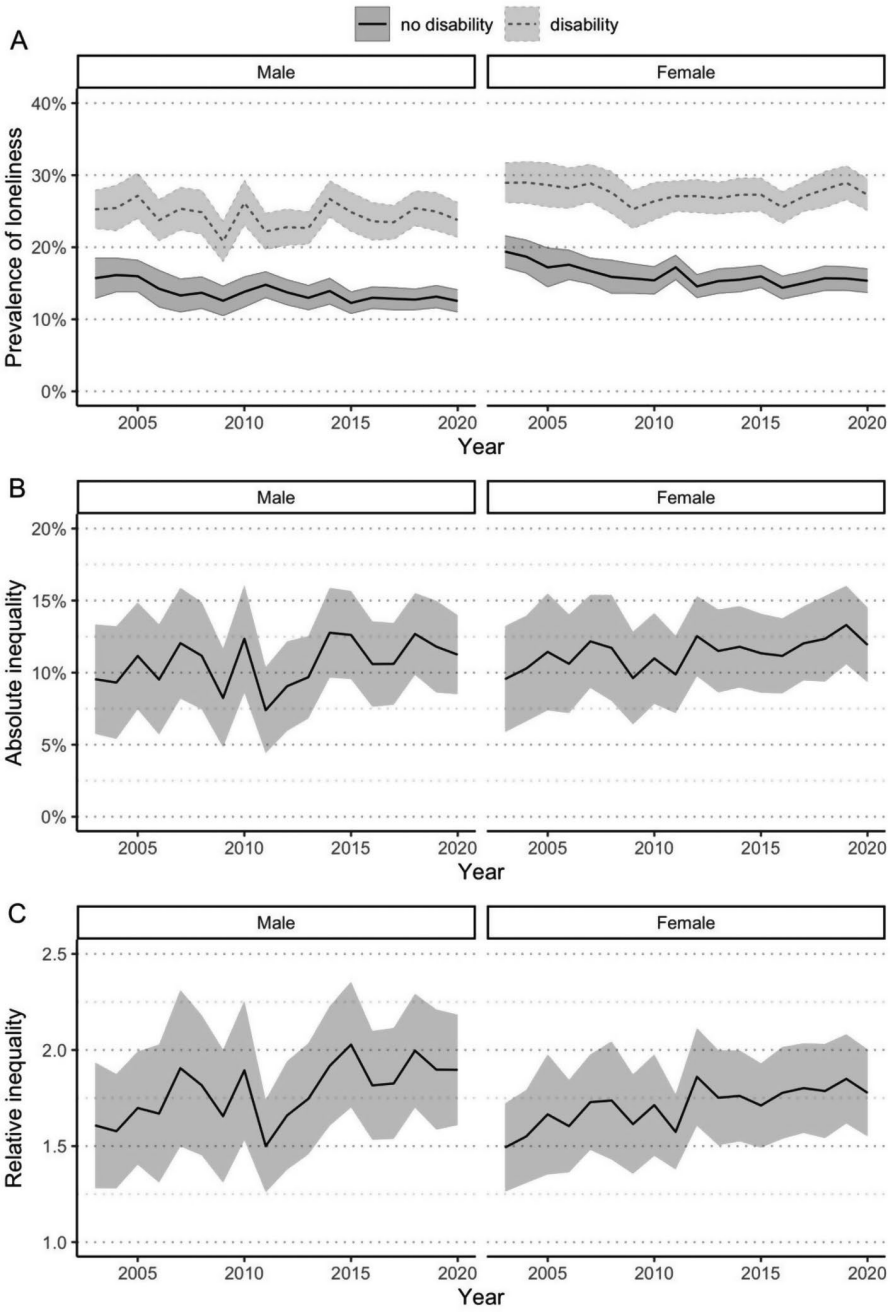
Loneliness over time for Australians aged 15+ years with and without disability, 2003–2020, stratified by sex. (**A**) Age-standardised population-weighted prevalence of loneliness over time, stratified by sex, with 95% confidence intervals. Absolute (**B**) and relative (**C**) inequalities in the prevalence of loneliness over time, stratified by sex, with 95% confidence intervals

Figure 3A shows that, when stratified by sex, the prevalence of loneliness was higher for both males and females with disability, but there was little difference in prevalence between males and females with disability. Furthermore, the prevalence of loneliness was unchanged between 2003 and 2020 for both males and females with disability. In contrast, the prevalence of loneliness for females without disability decreased from 19.4% in 2003 to 15.3% in 2020; however, it remained stable over time for males without disability. Figure 3B and C demonstrate that the absolute and relative inequalities in loneliness between people with and without disability were unchanged between 2003 and 2020, for both males and females.

### Loneliness prevalence and inequalities by disability group

**Fig. 4:**
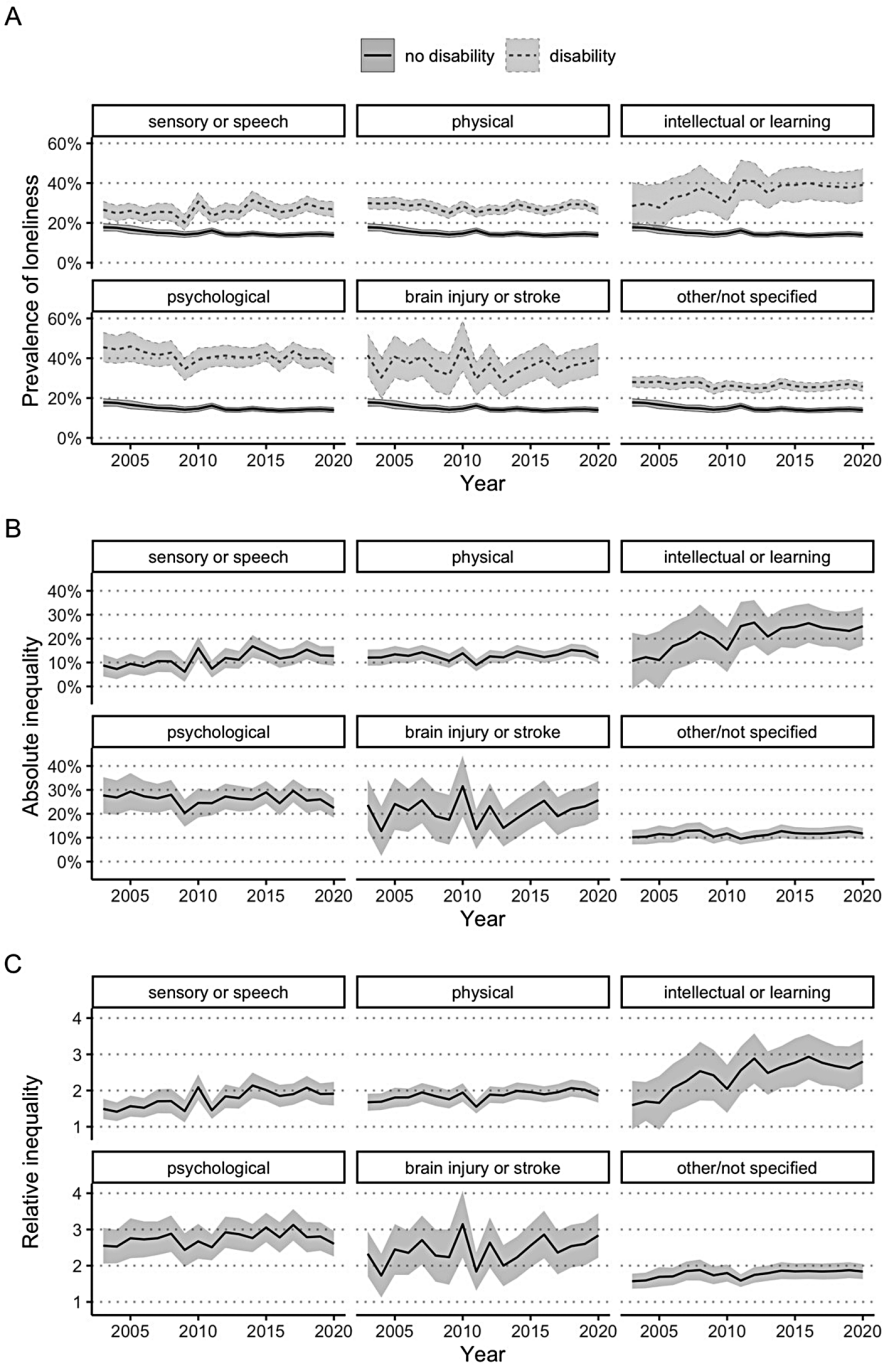
Loneliness over time for Australians aged 15+ years with and without disability, 2003–2020, stratified by disability group. (**A**) Age-standardised population-weighted prevalence of loneliness over time, stratified by disability group, with 95% confidence intervals. Absolute (**B**) and relative (**C**) inequalities in the prevalence of loneliness over time, stratified by disability group, with 95% confidence intervals. Note that individuals with disability may belong to more than one disability group

Figure 4A demonstrates that when stratified by disability group, the prevalence of loneliness was higher for all disability groups across the 18 years than it was for people without disability. In 2020, the prevalence of loneliness was considerably higher for people with intellectual or learning disability, psychological disability or brain injury or stroke compared to sensory or speech, physical, or ‘other’ disability. There was however no change in prevalence between 2003 and 2020 for any of the disability groups, given the overlapping 95% confidence intervals. Figure 4B and C demonstrate that the absolute and relative inequalities in loneliness for people in each of the disability groups, compared to people without disability, did not increase between 2003 and 2020. While there was a trend of increasing relative inequalities for people with intellectual or learning disability between 2003 and 2020, the 95% confidence intervals slightly overlapped. When compared with people without disability, the largest relative inequalities in loneliness in 2020 were observed for people with intellectual or learning disability (2.80 times more likely to experience loneliness), psychological disability (2.61 times more likely to experience loneliness), and brain injury or stroke (2.83 times more likely to experience loneliness).

## Discussion

This study found that for every year during the 18-year period from 2003 to 2020, the prevalence of loneliness was greater for people with disability, such that people with disability were 1.5 to 1.9 times more likely to experience loneliness than people without disability. These substantial and persistent inequalities in loneliness occurred regardless of sex, age or disability group. These sustained inequalities are of particular concern given that, although the prevalence of loneliness decreased for people without disability between 2003 and 2020, the prevalence of loneliness did not decrease for people with disability during this same period. Furthermore, inequalities in loneliness were more substantial for people with intellectual or learning disabilities, psychological disability, and brain injury or stroke, indicating that people in certain disability groups were more likely to experience loneliness.

Our findings that people with disability have increased risk of loneliness reinforce those from previous reports [17, 19–23] and adds to the existing evidence by demonstrating that inequalities in loneliness have persisted for decades without improvement. Loneliness is known to be associated with poorer physical and mental health outcomes [7–10] and is considered to be a public health concern in the general population [4, 5, 8, 9, 11, 12]; given this, the consistently higher levels of loneliness observed for people with disability may contribute to the considerable health inequalities experienced by this population [13, 15, 16]. In contrast, for people without disability loneliness prevalence decreased over the 18 years, mostly occurring between 2003 and 2009; yet there was no downward trend for people with disability over the same period. This difference in trends suggests potential differences in the drivers of loneliness, and their trends over time, between people with and without disability, which we did not examine in this study and requires further investigation.

We observed that the prevalence of loneliness and disability-related inequalities in loneliness were similar for males and females. We did however observe age differences in the prevalence of loneliness, such that younger people were more likely to experience loneliness than older people, particularly in the most recent survey years, with a possible increasing trend in prevalence developing for younger people (15–24 years) and a decreasing trend for older people (65+ years). This contrasts with the findings summarised in a recent meta-analysis of 57 studies between 2000 and 2019 suggesting that the prevalence of loneliness was higher in older adults than in young and middle aged adults; however, the authors of that meta-analysis also noted that the prevalence of loneliness varied considerably across countries and that the age pattern of loneliness may be context specific [4]. To our knowledge, ours is the first study to have assessed the prevalence of loneliness across a sample with a broad age range, for people with and without disability, and across an extended period of years. Importantly, our findings demonstrate that people with disability have considerable inequalities in loneliness compared to those without disability, and that this inequality decreases in older age groups (75+ years). This suggests that there may be a difference in the drivers of loneliness for people with disability after age 75, given that many will have developed disability as they aged alongside their peers.

To account for the diverse nature of disability, we undertook sub-analyses to examine how inequalities in loneliness vary by disability grouping. We observed that the substantial inequalities in loneliness for all six disability groups were sustained over the 18 survey years, however the relative inequalities were more pronounced for people with intellectual or learning disabilities, psychological disability, and brain injury or stroke, who were 2.6 to 2.8 times more likely to experience loneliness than people without disability. Furthermore, the relative inequalities appeared to increase by about 75% between 2003 and 2020 for people with intellectual or learning disabilities, however there was a slight overlap in confidence intervals because of the relatively low sample size in this group. Given that the present study is the first to assess loneliness across a range of disability groups over an extended period, the reason for greater inequalities within certain disability groupings is not clear. People with intellectual or learning disabilities, psychological disability, and brain injury or stroke experience higher levels of socio-economic disadvantage than people in other disability groups [14], which may explain their higher levels of loneliness given socio-economic disadvantage is a risk factor for loneliness [5].

This study has several strengths. First, we used a large, nationally representative sample of the Australian population over an 18-year period. The sample population had age distributions for people with and without disability that were mostly consistent with those observed for people with and without disability aged 15+ years in Australia’s national survey of disability, the SDAC [41]. Second, we employed direct age-standardisation using a reference population of people with disability, allowing us to address differences in age distribution between people with and without disability while ensuring that the prevalence of loneliness estimates reported reflect the reality for people with disability [37, 38]. Finally, the structure of the HILDA Survey disability question allowed us to compare the effects of loneliness on different disability groupings rather than only on the presence or absence of disability.

There were also limitations. First, the HILDA Survey focusses on people living within the community, thus people with severe disability who are more likely to reside in care homes and institutions are underrepresented in the sample. Second, selection bias may have occurred due to missing data, however multiple imputation was used to decrease potential bias. Third, statistical power was limited for some of the disability groupings that had small sample sizes (e.g., intellectual or learning disability and brain injury or stroke) and this may have obscured some of the trends due to large confidence intervals. Fourth, reporting bias in the measure of disability may have occurred given that disability information was self-reported. Reporting bias may also have occurred with the direct measure of loneliness used in the HILDA Survey given that the potential stigma of admitting to being lonely can alter estimates of the prevalence of loneliness when compared to indirect multi-question measures of loneliness that do not use the term ‘lonely’ [42, 43]. Nevertheless, the direct measure of loneliness used in this study has the advantage of solely assessing the subjective emotional experience of loneliness without being confounded by concepts associated with social isolation, support or trust that may be included in indirect multi-question measures. Fifth, we were unable to examine disability severity as a determinant of loneliness since the HILDA Survey only collects information on severity of disability and impact on daily activities every four waves. Sixth, the HILDA Survey is a panel survey, thus the outcome measure may be conditional on an individual’s experience in previous years; however, it is common for people to transition into and out of loneliness over the lifespan [44], thus while a person may experience loneliness in one wave, they may not be experiencing loneliness in the following wave. Seventh, while intellectual disability and learning disability are different types of disability, we were unable to distinguish between them in this study because the question prompt used in the HILDA Survey was “difficulty learning or understanding things”. Finally, the last wave of data (2020) was collected between 3 August 2020 and 21 February 2021, predominantly by telephone due to government-mandated social distancing requirements because of COVID-19; thus, the 2020 estimates of loneliness may have been impacted by COVID-19. However, for most subgroups, the prevalence of loneliness observed in the 2020 wave was consistent with the trends observed in the previous waves for those subgroups.

## Conclusions

Our findings indicate that addressing inequalities in loneliness for people with disability is a critical public health concern given that loneliness is not only a distressing experience that can cause considerable suffering [6], but is associated with a wide range of poor physical and mental health outcomes [7–10]. Loneliness is a multi-factorial societal problem thus interventions need to be disability inclusive and specifically address the loneliness experienced by people with disability. While population-level reductions in loneliness have been observed following whole-of-community interventions that build social connection [45] and the use of green spaces and contact with nature [46], solutions to reduce loneliness inequalities for people with disability need to directly address the disabling barriers that prevent people with disability from participating equally in society [e.g., 19] and their decreased social connectedness [18]. There is an urgent need to understand the drivers of loneliness for people with disability, including socio-economic and geographic factors and how these may change during the life course, to develop tailored interventions to address the inequalities in loneliness for people with disability reported in this paper. The next phases in this program of research will seek to: (i) gain a better understanding of the drivers of loneliness in people with disability, including those that cause loneliness to persist; (ii) examine how the experience of loneliness affects health outcomes for people with disability; and (iii) co-design policy interventions to create more equitable and welcoming communities to ameliorate the loneliness currently experienced by people with disability.

### Electronic supplementary material

Below is the link to the electronic supplementary material.

**Supplementary Material 1:** Additional methodological details for the analysis of missing data and the multiple imputation model. **Supplementary Table 1.** Observations that had missing values for the loneliness question, stratified by disability, demographic and socio-economic characteristics

## Data Availability

The data used are available free of charge to researchers through the Australian Data Archive (ADA) Dataverse, conditional on approval by the Director of Longitudinal Studies. HILDA General Release 20 (Waves 1–20) can be obtained here: https://dataverse.ada.edu.au/dataset.xhtml?persistentId=doi:10.26193/YP7MNU.

## Abbreviations

HILDA: Household, Income and Labour Dynamics in Australia Survey
ICF: International Classification of Functioning, Disability and Health
SDAC: Survey of Disability, Ageing and Carers
