# Supplementary material for: Disability-related inequalities in the prevalence of loneliness across the lifespan: trends from Australia, 2003 to 2020
Source: BMC Public Health. 2024 Feb 27;24:621. doi: 10.1186/s12889-024-17936-w (PMC10898179; doi:10.1186/s12889-024-17936-w)
Supplement: Supplementary file 1 — Supplementary Material 1: Additional methodological details for the analysis of missing data and the multiple imputation model. Supplementary Table 1. Observations that had missing values for the loneliness question, stratified by disability, demographic and socio-economic characteristics [file 12889_2024_17936_MOESM1_ESM.docx]

**Supplementary Materials**

**Methods**

*Analysis of missing data*

Of the 278,057 observations obtained during 2003 to 2020 from people aged 15+ years who responded to the disability question, 11.2% (n = 31,255) had missing values for the loneliness question. It should be noted that 90.7% of observations with missing values for the loneliness question resulted from participants not returning the Self-Completion Questionnaire that contained the loneliness question, while the remaining 9.3% are because participants refused to answer the question or provided invalid responses.

The proportion of observations with missing values for the loneliness question across the 18 waves varied from 8.7% to 14.5%. Table S1 shows the missingness of data for the loneliness question after stratification by disability, demographic and socio-economic characteristics. Participants who had missing responses to the loneliness question were more likely to be under 35 years or 75 years and above, male, had less than 12 years of education, unemployed, born outside of Australia in a non-English speaking country, not married or in a de-facto relationship, not living in a household as a couple. Furthermore, data for the loneliness question was more likely to be missing for participants who lived in areas with lower SEIFA quintiles (i.e., areas with greater socio-economic disadvantage), had lower life satisfaction scores, had intellectual or learning disabilities, or who had experienced a brain injury or stroke.

Data was also missing for < 0.1% of observations for the variables country of birth, relationship status, SEIFA (Socio-Economic Indexes for Areas) Index relative socio-economic disadvantage and life satisfaction, which were used for multiple imputation.

*Multiple imputation*

Table S1 shows the variables that were included in the imputation model. These variables were chosen to align with the conceptual model of loneliness proposed by Lim et al [1]. The imputation model used predictive mean matching based on the 5 nearest neighbours, with observations sampled by survey wave. This was done to ensure the values for the loneliness question were limited to the observed range of the scale (i.e., between 1 and 7) and preserved the non-normal distribution of the observed values. To accommodate for potential effects that may have been caused by changes in loneliness (and its determinants) over the 18 years, imputation used subsamples for each wave of the survey. For the < 0.1% of observations that had missing values for the variables used to impute loneliness, imputations were ‘forced’ so that only complete variables were used. The population-weighted estimates for each wave were determined for each of the 50 imputed datasets, from which an overall estimate of the proportion of people experiencing loneliness was obtained. Rubin rules for combining the between-imputation and within-imputation variance were used to derive standard errors, obtained from the Taylor Series linearisation standard errors of the loneliness estimates.

**References**

1. Lim MH, Eres R, Vasan S (2020) Understanding loneliness in the twenty-first century: an update on correlates, risk factors, and potential solutions. Social Psychiatry and Psychiatric Epidemiology 55 (7):793-810. doi:10.1007/s00127-020-01889-7

**Supplementary Table 1.** Observations that had missing values for the loneliness question, stratified by disability, demographic and socio-economic characteristics

|  | Number of observations with missing values for the loneliness question | % of observations with missing values |
| --- | --- | --- |
| Analytic sample ^a^ | 31,255 | 11.2% |
| Disability |  |  |
| No disability | 21,986 | 11.0% |
| Any disability | 9,269 | 11.8% |
| Age (years) |  |  |
| 15-24 | 7,416 | 15.0% |
| 25-34 | 7,194 | 14.7% |
| 35-44 | 5,081 | 10.9% |
| 45-54 | 4,066 | 8.8% |
| 55-64 | 2,644 | 6.8% |
| 65-74 | 1,845 | 6.7% |
| 75+ | 3,009 | 14.7% |
| Sex |  |  |
| Male | 16,155 | 12.3% |
| Female | 15,100 | 10.3% |
| Education |  |  |
| Completed Year 12 | 19,555 | 10.2% |
| Not completed Year 12 | 11,700 | 13.7% |
| Employment |  |  |
| Employed | 19,329 | 11.0% |
| Unemployed | 1,705 | 15.8% |
| Not in labour force | 10,221 | 11.3% |
| Country of birth |  |  |
| Australia | 23,735 | 10.8% |
| Other English-speaking country | 2,155 | 8.3% |
| Other non-English speaking country | 5,360 | 16.5% |
| Married/de-facto relationship |  |  |
| Yes | 16,176 | 9.4% |
| No | 15,066 | 14.3% |
| Household |  |  |
| Couple, with/without children, with/without other adults | 18,341 | 9.5% |
| Lone parent, with children, without other adults | 3,329 | 15.1% |
| Lone parent, with children, with other adults | 628 | 17.2% |
| Lone person | 6,756 | 15.0% |
| Person not part of a couple, with other adults | 2,201 | 15.9% |
| SEIFA Index (relative socio-economic disadvantage) |  |  |
| 1^st^ quintile (lowest) | 7,370 | 14.4% |
| 2^nd^ quintile | 6,260 | 11.6% |
| 3^rd^ quintile | 6,296 | 11.2% |
| 4^th^ quintile | 6,197 | 10.4% |
| 5^th^ quintile (highest) | 5,120 | 9.1% |
| Life satisfaction |  |  |
| 0 (completely dissatisfied) | 80 | 22.8% |
| 1 | 85 | 18.7% |
| 2 | 184 | 18.0% |
| 3 | 340 | 17.1% |
| 4 | 506 | 15.8% |
| 5 | 1,827 | 17.1% |
| 6 | 2,224 | 13.8% |
| 7 | 6,181 | 11.7% |
| 8 | 10,014 | 10.6% |
| 9 | 5,547 | 8.9% |
| 10 (completely satisfied) | 4,163 | 12.4% |
| Disability group ^b^ |  |  |
| Sensory or speech | 2,514 | 13.1% |
| Physical | 5,566 | 11.6% |
| Intellectual or learning | 931 | 22.8% |
| Psychological | 1,862 | 13.3% |
| Brain injury or stroke | 550 | 17.8% |
| Other/type not specified | 5,334 | 11.7% |

*^a^ Includes people aged 15+ years, who responded to the disability question, who were interviewed during in waves 3 to 20 (2003 to 2020).*

*^b^ Only available for people with disability, who may be included in more than one disability group.*
